# Health system performance on greenhouse gas emissions, climate change and development status in 38 OECD countries

**DOI:** 10.1038/s41598-025-89485-0

**Published:** 2025-02-11

**Authors:** Jeffrey Braithwaite, Yvonne Tran, Georgia Fisher, Louise A Ellis, Carolynn L Smith, Yvonne Zurynski

**Affiliations:** 1 Centre for Healthcare Resilience and Implementation Science, Australian Institute of Health Innovation, Macquarie University, 75 Talavera Road, North Ryde, Sydney, 2113 Australia; 2 NHMRC Partnership Centre for Health System Sustainability, Macquarie University, 75 Talavera Road, North Ryde, Sydney, 2113 Australia; 3 International Society for Quality in Health Care, Suite G01, 48 Mount Street Upper, Dublin, D02 YY23 Dublin 2 Ireland; 4 Observatory on the Future of Healthcare, Australian Institute of Health Innovation, Macquarie University, 75 Talavera Road, North Ryde, Sydney, 2113 Australia; 5 Hearing Research Centre, Macquarie University, 75 Talavera Road, North Ryde, Sydney, 2113 Australia

**Keywords:** Climate change, Health systems, OECD, Climate adaptation, Climate mitigation, Cross-country comparisons, Climate sciences, Climate change, Health care, Health policy, Health services, Public health

## Abstract

Health systems must solve two climate-related problems simultaneously: mitigate their greenhouse gas emissions and adapt to additional patient loads associated with climate-induced demands for care from weather sequalae and vector-borne diseases. We aimed to benchmark the mitigation and adaptive efforts of OECD member countries compared with their health system performance. We used Multidimensional Scaling and Hierarchical Cluster Analysis to group all 38 OECD member countries as at January 2024. Clusters were defined according to greenhouse gases per capita, vulnerability to climate change and the United Nations’ Human Development Index. Post hoc analyses compared each cluster’s performance to the OECD’s Health Care Quality Indicators. The countries fell into five profiles: Vulnerable low emitters; Vulnerable aspiring; Middle of the road; Robust aspiring; and Goldilocks zone. Post-hoc analyses revealed strong links between the profiles and health system performance. Characterising countries in a five-dimensional model may help policymakers to share benchmarked country-level information, devise ways to support resilient care practices, better target healthcare investments, and improve access to needed care.

**Supplementary Information:**

The online version contains supplementary material available at 10.1038/s41598-025-89485-0.

## Introduction

**Fig. 1:**
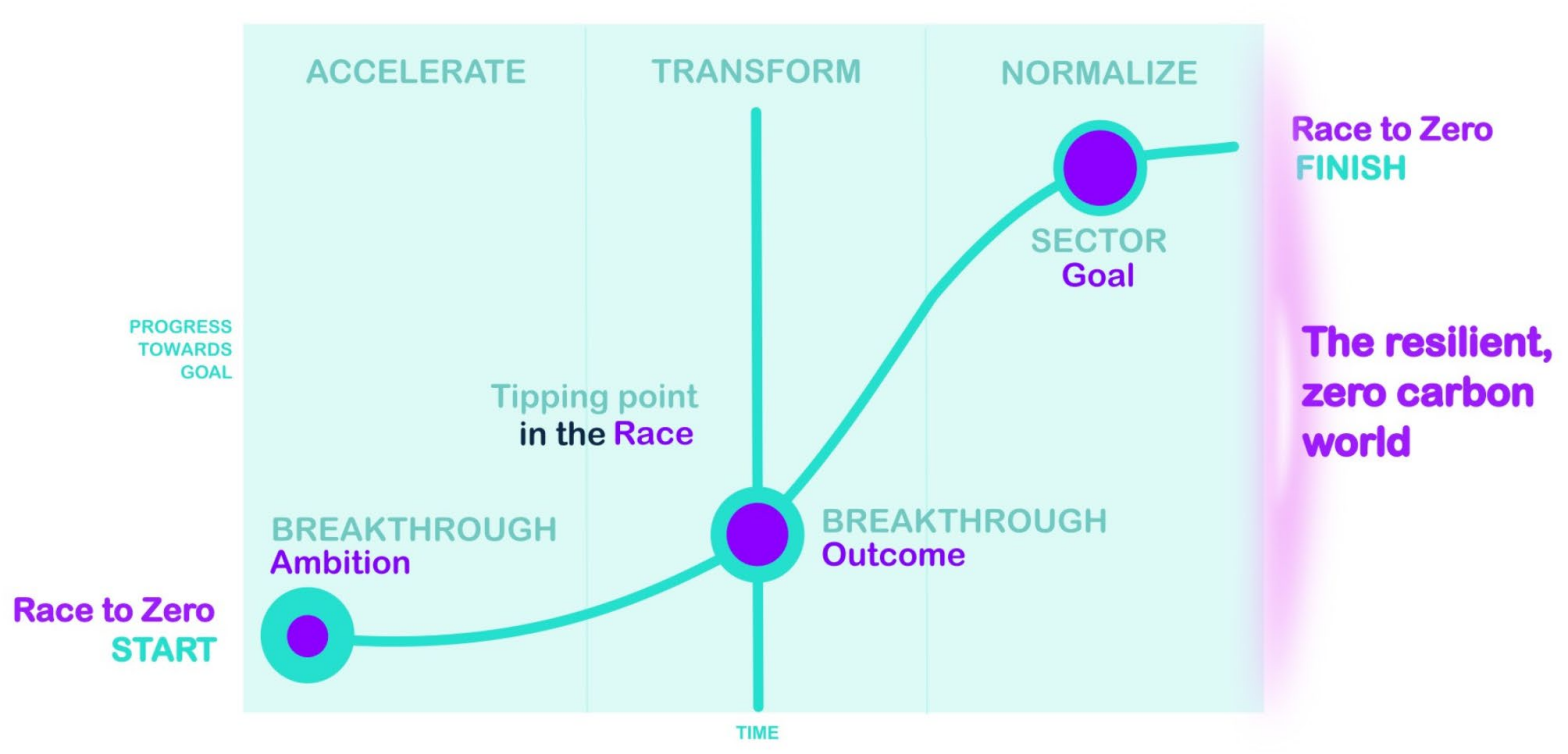
Overarching considerations in getting to net zero. Source: Adapted from United Nations Framework Convention on Climate Change^6^.

There are times when it seems the race to achieve net zero carbon emissions will fail, with many in the world responding with anxiety, depression, and especially amongst many young people, existential dread^1–3^. However, there are also moments when international bodies and state actors respond positively to humankind’s greatest challenge, providing reasons for optimism^4,5^. Ideally, the race to net zero should follow the trajectory in Fig. 1 below. Analysing the current progress of the member countries of the Organisation for Economic Co-operation and Development (OECD) presents an unparalleled opportunity to assess where we are at a country level in the net zero journey.

**Fig. 2:**
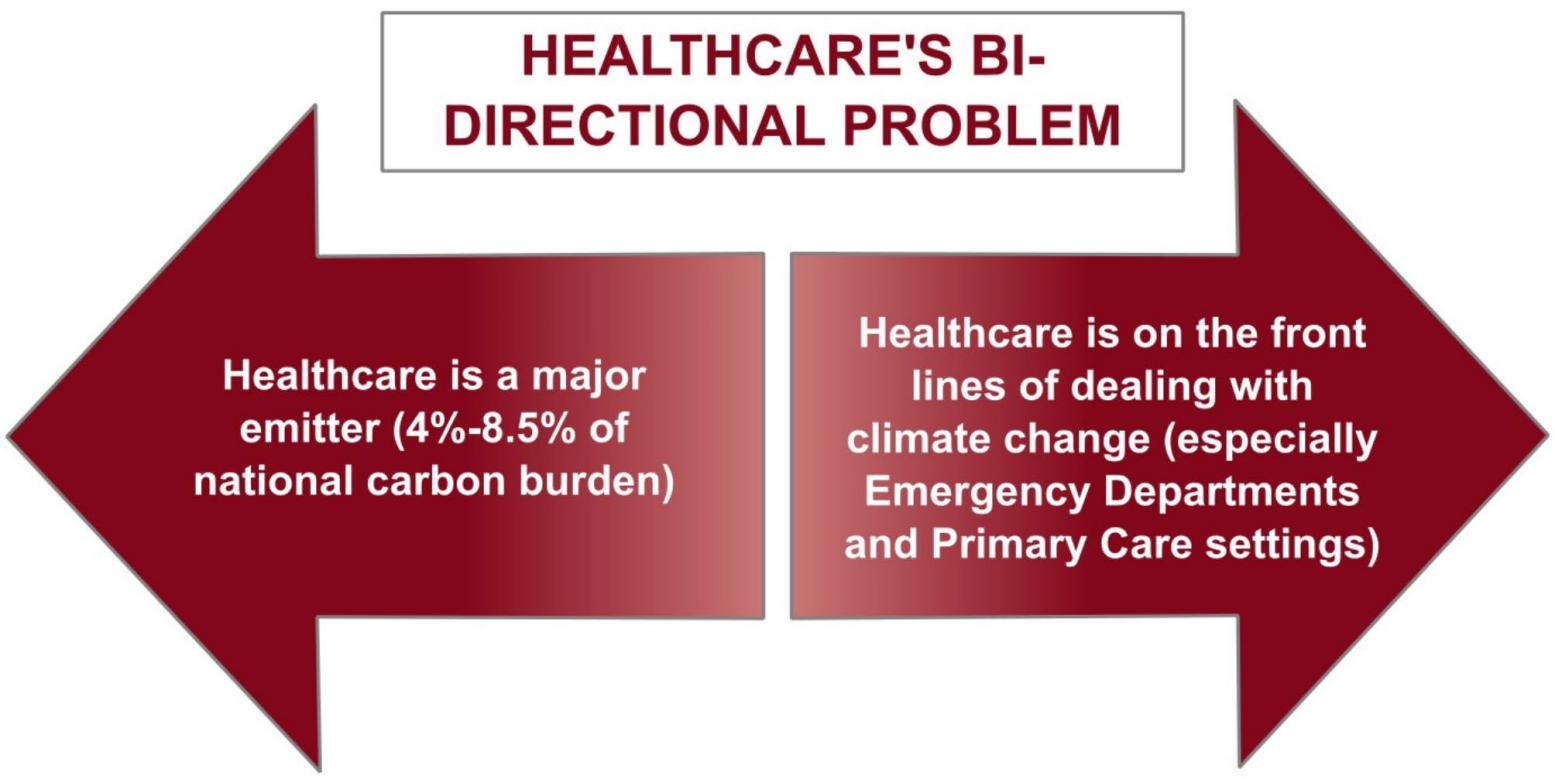
Healthcare’s unique challenge. Source: Authors’ conceptualisation.

As we consider the global efforts and challenges in mitigating climate change, it is important to examine specific sectors, especially those that have a large carbon footprint or are challenged in having to adapt under pressure. Health systems simultaneously contribute to greenhouse gas (GHG) emissions (GHGe) and must respond to threats to human health as a consequence of climate impacts^7–12^. The question we are asking is: *Can we create a benchmarking system for OECD countries that can provide insights into the factors that will affect their health system’s progress on mitigating their GHG emissions and adapting to climate change??* This sector, responsible for more than 4% of total global GHGe, also plays a crucial role on the front lines of care, providing increasingly needed services in response to climate-induced harm. As such, healthcare’s climate related challenges are bi-directional (Fig. 2).

Climate-change related harm to patients—from increasingly frequent and severe heat waves, wildfires and wildfire-induced particulate matter, flooding, droughts, and shifting patterns of tropical diseases—is set to increase substantially as we enter what United Nations Secretary-General António Guterres termed in mid-2023 the ‘era of global boiling’^13^. Globally, over five million deaths per year are associated with extreme temperatures^14^. Rising temperatures are also linked to immediate health impacts, such as heat exhaustion, and can contribute to the onset or exacerbation of longer-term health conditions, such as kidney disease, asthma, and chronic obstructive pulmonary disease (COPD).

These health consequences directly affect not only populations, regions, local communities, families and individuals, but the systems that care for them. Health systems are witnessing increases in the volume and complexity of care needed. The urgency of this situation cannot be overstated. Health systems must adapt to become fit-for-purpose and capable of reducing their own GHGs as an industry, while continuing to deliver quality care to all. A health system’s capacity to mitigate its GHG emissions and to adapt to the effects of climate change will in part be dependent on country level factors. For example, most of health system’s carbon footprint arises from their energy usage and their supply chains^15^. Health systems in countries that have policies and laws committing to net zero and have begun to decarbonise their energy sector may benefit from these country level actions in their own journey to net zero^15^. More developed countries and ones that have stronger infrastructure or have taken steps to prepare for the impacts of climate change (e.g., through early warning systems linked to health systems) may also support their health system’s adaptive capacity. The current level of health system performance is in part determined by these factors, but will also reflect their resilience to shocks created by extreme weather events and capacity to adopt mitigation actions.

Here, we build on earlier studies tracing regional- and country-level progress with a deeper understanding of healthcare’s challenges. Andrieu and colleagues^16^ examined resource consumption in 49 regions of the world, benchmarking that against the regional populations’ access to, and quality of, care. They raised concerns about healthcare’s dependence on fossil fuels and the challenges in becoming more efficient, providing universal coverage, and maintaining high-quality care.

## Aims

Despite the valuable contributions of previous studies, no one has examined the relationship between OECD countries’ GHG emissions, vulnerability to climate change and their development status to create country profiles to understand the external context in which health systems operate as they attempt to address their bi-directional problem. We aimed to consider the relationship between these country level profiles and their health systems’ performance to create a benchmarking system. Our benchmarking system situates countries within a matrix of factors that affect health systems’ capacity to adapt and respond to climate change, which can then be used to guide future policy, monitoring, and responsiveness. This is the 38-OECD Countries’ Health System Performance (CHSP) Green Study.

## **Methods**

### Sampled countries

We included a census of the 38 OECD member countries at January 2024: Australia, Austria, Belgium, Canada, Chile, Colombia, Costa Rica, Czech Republic, Denmark, Estonia, Finland, France, Germany, Greece, Hungary, Iceland, Ireland, Israel, Italy, Japan, Korea, Latvia, Lithuania, Luxembourg, Mexico, Netherlands, New Zealand, Norway, Poland, Portugal, Slovak Republic, Slovenia, Spain, Sweden, Switzerland, Türkiye, United Kingdom (UK), and United States of America (USA).

### Measures

We examined the relationship between countries’ GHGe, vulnerability to climate change, and their development status. An assessment was also made of relative health system performance, yielding 342 composite scores in total across the data sets.

GHGe per capita datasets were obtained from the OECD Environment Statistics database^17^, a policy-relevant collection of key information. This collection holds data on human-made gases. CO_2_ is not the only gas driving global climate change, but others—methane, nitrous oxide, and trace gases, such as the group of ‘F-gases’—are all contributors to emissions. Higher numbers indicate more GHGe.

Vulnerability to climate change was assessed using the Notre Dame Global Adaptation Initiative (ND-GAIN) index^18^. The ND-GAIN open-source index shows a nation’s current vulnerability to climate disruptions. It comprises two indices: the Vulnerability Index (comprising 36 indicators) and the Readiness Index (comprising 9 indicators). For this study we used the Vulnerability Index, which considers six life-supporting service groups (each represented by six indicators): food, water, health, ecosystem services, human habitat, and infrastructure. The Vulnerability Index ranges from 1 to 100, with higher ratings indicating higher levels of vulnerability.

The Human Development Index (HDI) is the United Nations’ (UNs’) measure of average achievement on key dimensions of people and their inherent and actual capabilities and capacities^19^. It serves as a composite indicator of development status. The HDI considers three dimensions and is a mean of normalised indices of these three dimensions: a long and healthy life; education levels; and decent living standards. The values of the HDI range from zero to one, with higher values signifying higher levels of human development.

Data pertaining to health system performance were sourced from the OECD Health Care Quality Indicator Project, the ‘Health at a Glance 2021’ framework^20^. This framework offers a collection of analytical dashboards that assess the effectiveness of OECD nations in fostering population health and enhancing health system operations. These dashboards provide a concise comparison of OECD members based on key health performance metrics, helping pinpoint areas requiring attention. Specifically, the dashboard data gauges the performance of countries across six key health dimensions: (1) Health Risk Factors, (2) Health Status, (3) Access to Healthcare, (4) Quality of Healthcare, (5) Health System Resources and (6) COVID-19 Factors. Example indicators for Health Risk Factors include smoking prevalence, alcohol consumption, obesity rate and environmental risk factors such as air pollution. For Health Status, indicators include life expectancy, avoidable mortality rates, prevalence of chronic diseases (e.g., diabetes, heart disease), and self-reported health status. Access to Healthcare is measured by indicators such as service coverage rate, proportion of the population with coverage by necessary health services, and protection from financial hardship. Quality of Healthcare indicators encompass patient satisfaction scores, the rate of antibiotic prescribed, avoidable chronic obstructive pulmonary disease, mammography screening and mortality following acute myocardial infarction. Health System Resources are gauged by the number of healthcare professionals per capita (e.g., doctors, nurses), hospital bed availability, and healthcare expenditure. COVID-19 Factors are measured by the COVID-19 vaccination rate, COVID-19 infection rate, and COVID-19 mortality rate.

Standard z-scores were computed for each health dimension, with individual scores determined for each metric. Since each health dimension encompasses a minimum of four indicator metrics, the mean of these standard scores was taken to represent the aggregate score for the respective health dimension.

### Data analysis

#### Country cluster identification

We employed Multidimensional Scaling (MDS) and Hierarchical Cluster Analysis to graphically represent the relationships among enrolled countries, focusing on their GHGe per capita, vulnerability to climate change, and HDI. MDS is particularly useful in this context as it allows us to visualise the similarities or differences in data by positioning each country in a two-dimensional space based on the calculated distances between them. This method helps identify underlying patterns in complex data sets by simplifying multidimensional data into more comprehensible two or three-dimensional plots^21^.

For the Hierarchical Cluster Analysis, we utilised the scipy.cluster.hierarchy Python package (version 1$\:.$13$\:.$0). This method enabled us to construct a dendrogram that illustrates how closely countries are related to each other based on the GHGe, vulnerability to climate change, and HDI dimensions. We applied the Ward linkage method, which minimises the variance within each cluster, ensuring a more coherent and meaningful grouping^22^. This analysis led us to determine an optimal grouping into five distinct clusters. We employed goodness-of-fit statistics to refine our MDS model further and ensure that the dimensional reduction retained the essential characteristics of our multidimensional data. This was conducted using the sklearn.manifold package (version 1$\:.$4$\:.$1$\:.$post1). The goodness-of-fit measure allowed us to select the number of dimensions that best represented the data’s structure, enhancing the interpretability and accuracy of the spatial representation.

#### Country cluster comparison

 To examine the health systems’ performance against the country profiles, one-way ANOVA with post-hoc comparisons were made against the “Health at a Glance” dimension aggregate scores. This facilitated an assessment of progress on population healthiness and health systems performance across the OECD countries.

### Role of funding source

The funder of the study had no role in study design, data collection, data analysis, data interpretation, or writing of the report.

## Results

**Fig. 3:**
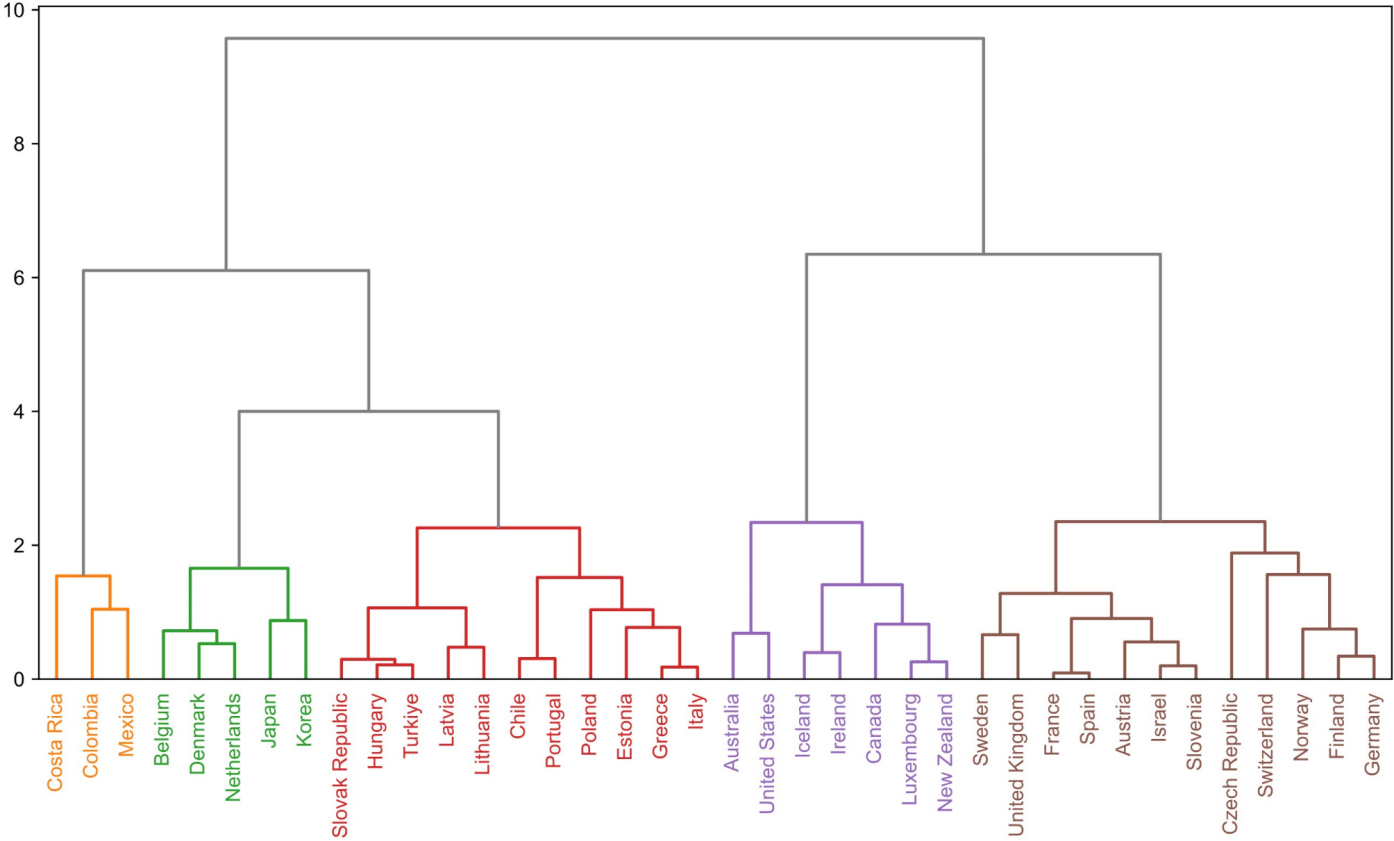
Dendrogram results from the hierarchical cluster analysis.

**Fig. 4:**
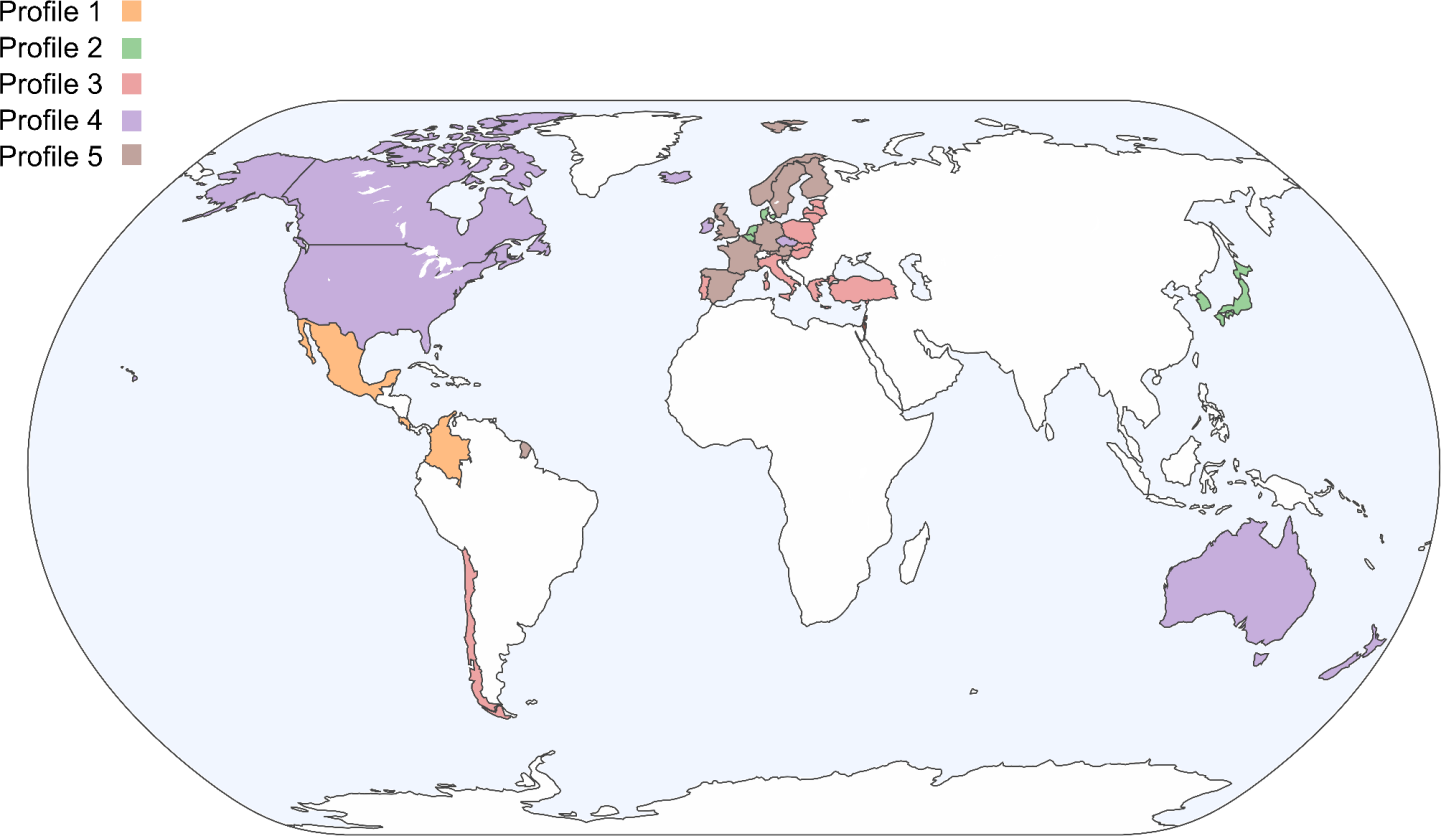
The distribution of countries within each of the five profiles. Map of countries included in our analysis. Figure was created with the Python programming language, using the package geopandas version 1.0.1 and coloured according to their assigned Profile.

Five clusters were identified to be the optimal number of groups into which the 38 countries fell. Figure 3 shows the dendrogram results of the hierarchical analysis. Five clusters were identified when the branches were cut at height three, with each cluster representing a profile of progress in healthcare’s bidirectional climate problem. Profile 1 (yellow) comprised three countries: Costa Rica, Colombia, and Mexico. Profile 2 (green) consisted of five countries: Belgium, Denmark, Netherlands, Japan, and Korea. Profile 3 (red) contained 11 countries: Slovak Republic, Hungary, Türkiye, Latvia, Lithuania, Chile, Portugal, Poland, Estonia, Greece, and Italy. Profile 4 (purple) grouped seven countries: Australia, United States, Iceland, Ireland, Canada, Luxemburg, and New Zealand. Profile 5 (brown) the largest group, contained 12 countries: Sweden, United Kingdom, France, Spain, Austria, Israel, Slovenia, Czech Republic, Switzerland, Norway, Finland, and Germany (Fig. 4).

**Figs. 5:**
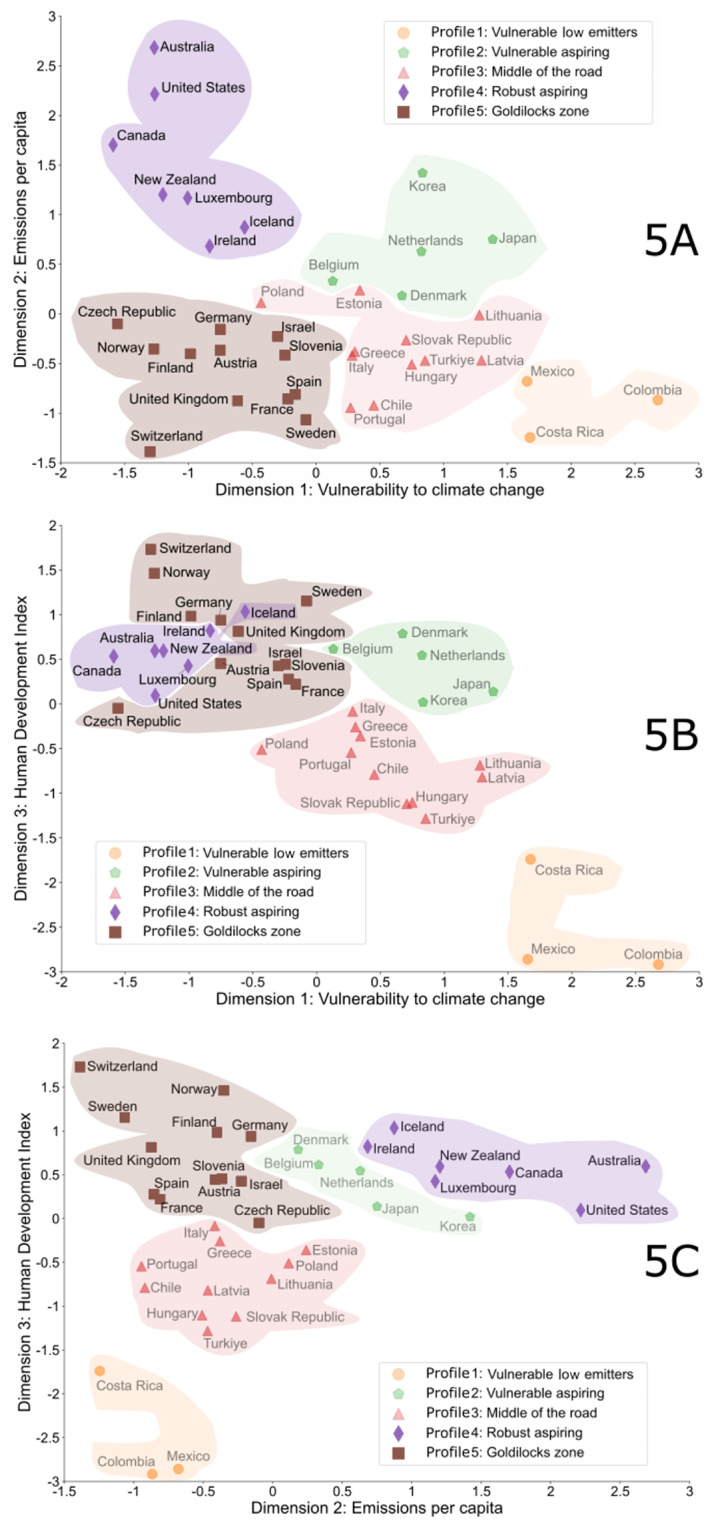
(**a**–**c**) Relative clustering of OECD countries on three dimensions.

**Fig. 6:**
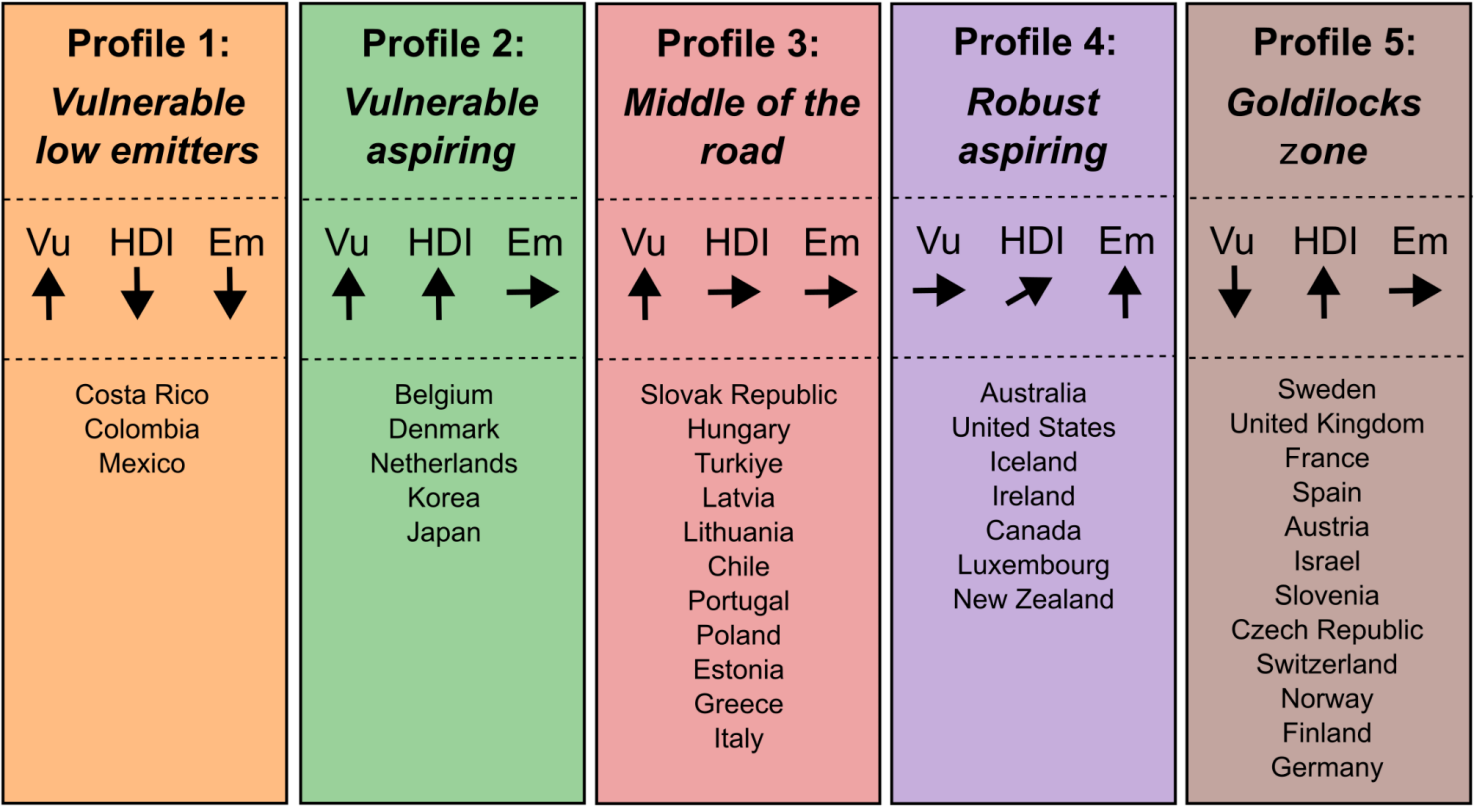
OECD country clusters.* VU* vulnerability index,* HDI* human development index,* Em* GHG emissions per capita.

The goodness-of-fit statistics confirmed the best fit variables as (1) Vulnerability, (2) GHGe, and (3) HDI. Figure 5a and c each illustrate cluster performance in these dimensions. Figure 6 summarises the data, with an explanatory label reflecting the countries’ status across the variables.

Regarding health systems performance for the five profiles, there were significant differences in dimension scores of Health Risk Factors (F(4,33) = 7$\:.$88, *p* < 0$\:.$001), Health System Resources (F(4,33) = 13$\:.$14, *p* < 0$\:.$001), Health Status (F4,33) = 7$\:.$18, *p* < 0$\:.$001), Quality of Healthcare (F(4,33) = 6$\:.$39, *p* < 0$\:.$001) and Access to Healthcare (F(4,33) = 14$\:.$89, *P* < 0$\:.$01). There were also differences in COVID-19 factors (F (4,33) = 3$\:.$46, *p* = 0$\:.$018).

**Fig. 7:**
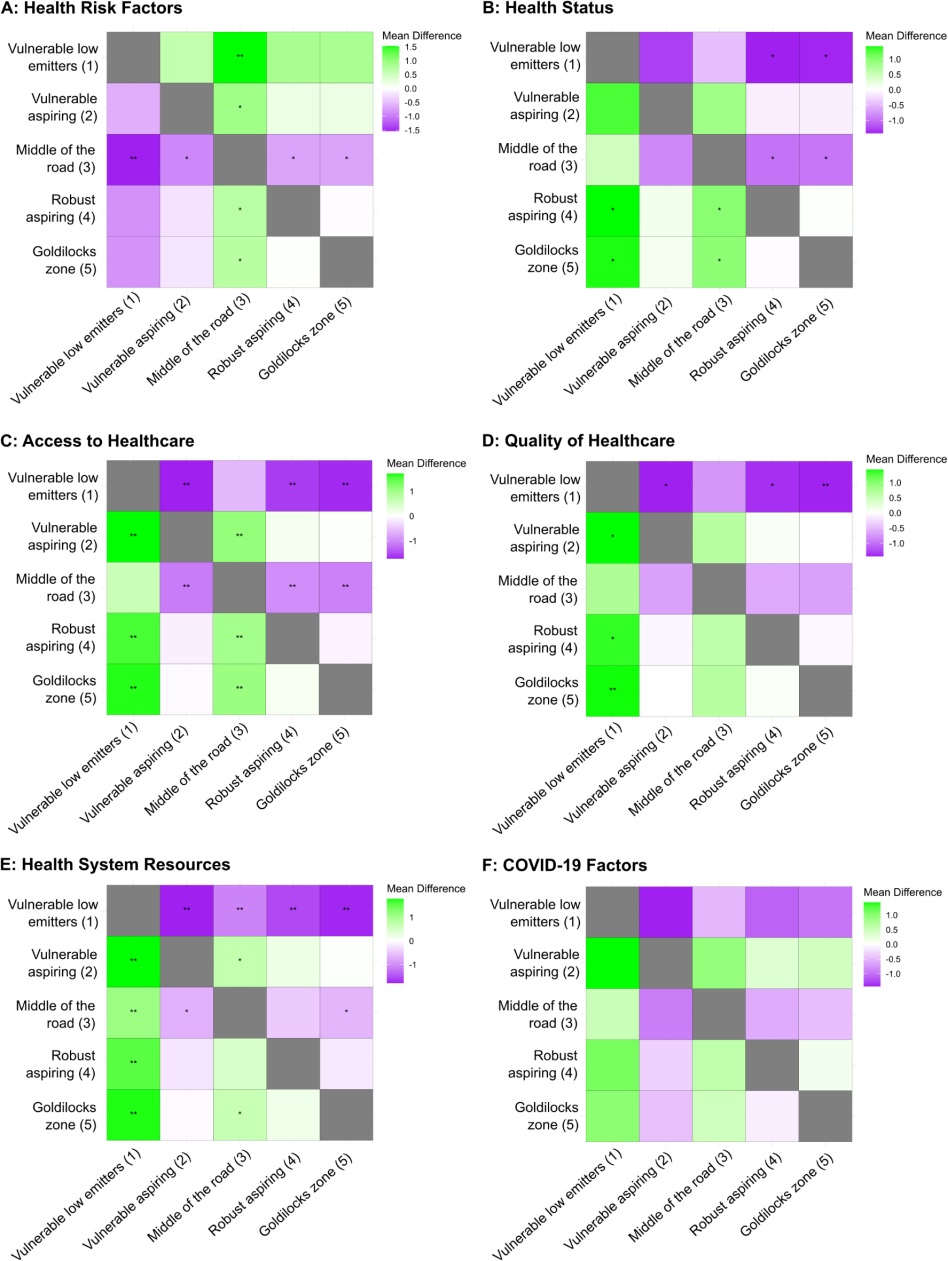
(**a**−**f**) Heatmaps of mean differences between country profiles and health system performance indicator. Colours indicate mean difference between country profiles based on health system performance indicator. *indicates statistically significance difference of < 0.05 after Bonferroni correction. **indicates statistically significance difference of < 0.01 after Bonferroni correction.

Post-hoc analyses applying a Bonferroni correction were conducted (Tables S1-S6 in Supplementary Materials). For the Health Risk Factors dimension score, there were significant differences between Profile 3 and the other four profiles, showing greater health risks in this profile. For Health System Resources, Profile 1 was significantly different compared to the other four profiles showing significantly less health capacity. Profile 3 was also significantly lower in Health System Resources than Profiles 2 and 5. For Health Status, Profile 1 and Profile 3 were both significantly lower compared with Profiles 4 and 5. Quality of Healthcare was significantly lower for Profile 1 than the other four profiles. Access to Healthcare was also comparable between Profiles 1 and 3 and both profiles were rated lower in access to care compared to Profiles 2, 4 and 5. For the COVID-19 Factors score, most of the Profiles were not significantly different; the main difference was found between Profile 1 and 2 (with a p-value of 0$\:.$05) (Fig. 7a and f).

## Discussion

In recent years, literature has proliferated that describes the contribution of health systems on climate change^10,11^, their level of preparedness for the impacts of climate change^12^, and the strategies that could be used to address each of these issues^7,8,10^. As climate change progresses, it will be essential to concurrently assess the world’s progress towards adaptation and resilience to the health system impacts of climate-change. Our study is a step forward, providing an initial benchmark of such an assessment for OECD member countries, namely, their vulnerability to climate change, development status, and GHGe.

In discussing these findings, we make six observations on health systems performance as they relate to the country profiles established in the MDS results: Health Risk Factors; Health System Resources; Health Status; Quality of Healthcare; Access to Healthcare; and COVID-19 Factors. By integrating the five health performance indices with the OECD country clusters, we can begin to identify the key factors that support or inhibit health systems’ capacity to address the bi-directional challenges posed by climate change. These interactions highlight the importance of contextualising health system performance within broader country profiles to understand better the pathways through which mitigation and adaptation efforts can be optimised.

### Health risk factors

Countries in Profile 3, Middle of the road, exhibited significantly greater health risks than the other profiles. The elevated health risks in Profile 3 could be attributed to a combination of lifestyle factors and environmental exposure. This suggests that despite a middle-ground positioning in emissions, vulnerability and HDI, these countries face notable challenges in health-related risk factors that may warrant targeted health interventions.

When examining differences between profiles, several patterns emerge. The Middle of the road profile encompasses countries with significantly higher vulnerability than Profiles 2, 4 and 5, indicating greater exposure to climate-related risks such as extreme weather events or environmental degradation. This elevated vulnerability likely compounds their health risks, straining health systems already challenged by limited resources. Compared to Profile 2, Profile 3 also exhibits lower HDI, suggesting that even among countries with moderate emissions, gaps in development contribute to their higher health risks. These differences suggest that the potential value of interventions that address systemic vulnerabilities and the socio-economic constraints unique to Profile 3 countries (Fig. 7a).

### Health system resources

Profile 1, Vulnerable low emitters, containing Colombia, Costa Rico and Mexico, provided significantly fewer health system resources than other profiles. Profile 1’s lower levels of resources may indicate systemic issues in less well-endowed economies, and the provision of insufficient healthcare funding, shortages in healthcare workers, or inadequate infrastructure. This lack of capacity is critical as it may hamper these countries’ abilities to respond effectively to climate-induced health crises. Conversely, the Goldilocks zone countries of Profile 5 demonstrate more robust health system resources aimed at contributing to better health outcomes. Profile 5 countries to a considerable extent exhibit higher HDI, which enables more substantial investments in health infrastructure, workforce development, and preparedness measures. This combination of lower vulnerability and higher HDI positions these countries to manage better and adapt to the dual challenges of climate change and health system demands. The stark contrast between these two profiles shows the importance of considering systemic capacity and contextual vulnerabilities, especially when considering strategies for strengthening climate resilience and health systems (Fig. 7b).

### Health status

Profiles 1 and 3’s lower population health status highlights a critical need for interventions that address both acute and chronic healthcare provision. These profiles, which include countries with either extreme vulnerability or middle-of-the-road performance, had significantly lower health status than Profile 4, Robust aspiring and Profile 5, Goldilocks zone. This indicates disparities in health outcomes that could be linked to socioeconomic factors, public health infrastructure, or investment levels, for example.

Climate mitigation and adaptation strategies adopted by countries from Profile 5 have critical implications for environmental sustainability and for their health status. Effective climate policies, such as decarbonising energy sectors, improving air quality, and enhancing resilience to climate-induced events, directly contribute to better health outcomes by reducing exposure to pollutants, preventing heat-related illnesses, and mitigating the health impacts of extreme weather events. The United Kingdom is a good example of a Profile 5 country with strong environmental policies^15,23^.

Conversely, countries with lower population health status, such as those in Profile 1, face additional headwinds. A lower health status contributes to a higher burden on the health system, increasing demand for healthcare services and resources. This elevated demand strains system capacity making it more challenging for these countries to mitigate emissions, even if emission reduction policies have been adopted, as health systems must prioritise immediate care needs over financial investments in emission reduction activities. Countries with limited resources or higher vulnerability must navigate the dual challenge of addressing immediate health needs while also working to reduce their environmental impact (Fig. 7c).

### Quality of healthcare

The significantly lower levels of quality of care in Profile 1 compared to other profiles (i.e., 2, 4, and 5) is of concern and signals the need for quality improvement initiatives. The quality-of-care concerns within Profile 1 can significantly affect patient outcomes and overall system efficiency. This may reflect underlying issues such as underfunding, lack of medical resources, or workforce challenges.

The observed differences in quality of care between Profile 1 and other profiles may be partially driven by disparities in HDI levels. Given that HDI is a proxy for the overall resources and infrastructure, Profile 1’s relatively lower HDI suggests more limited resources available for investment in health infrastructure, workforce development, and service delivery quality. This, in turn, points to reduced health system capacity to adapt to the impacts of climate change, as the ability to respond to climate-related health challenges often depends on a system’s baseline quality, efficiency, and resource availability (Fig. 7d).

### Access to healthcare

Access to care was significantly lower for Profiles 1 and 3 than for the more affluent Profiles 2, Vulnerable aspirers, 4, Robust aspiring, and 5, Goldilocks zone. These results highlight equity disparities across the OECD, where economic and social factors may create barriers to accessing health services. Countries in Profiles 1 and 3 typically have lower HDI and higher climate vulnerability, indicative of more constrained economic resources, reduced educational attainment, and limited health infrastructure. Higher vulnerability means these countries are more exposed to the adverse effects of climate change, which strain already under-resourced health systems. These constraints often translate into challenges such as shortages of healthcare providers, uneven distribution of health facilities, and financial barriers that limit individuals’ ability to obtain necessary health services. These issues reduce the overall availability and accessibility of care, leaving populations in these countries at greater risk of unmet health needs and worsening health outcomes. In contrast, Profiles 2, 4, and 5 generally benefit from higher HDI scores, which correlate with stronger economies, better-educated populations, and more robust healthcare (Fig. 7e).

### Covid-19 factors

The COVID-19 pandemic highlighted the importance of health system resilience^24^. The fewer number of significant differences in COVID measures between most profiles may reflect the globalised response to the pandemic, where the international sharing of resources and knowledge has been unprecedented. However, the borderline significant notable difference between Profiles 1 and Profile 2 suggests differential impacts of the pandemic, likely influenced by variations in health system resilience, political responses, and preparedness (Fig. 7f).

### Implications for policy and practice

The observed disparities highlight the importance of tailored health policies that address specific weaknesses within each profile group. Investments in health systems, addressing social determinants of health, and improving healthcare delivery can be vital for vulnerable profiles as climate increasingly affects communities and more sustained healthcare is needed. The relatively better performance of the Goldilocks zone countries can serve as a benchmark for others. The countries in the Goldilocks zone demonstrate what is possible with a sound balance of resources (such as universal healthcare coverage) and policies (such as net zero emission policies). There is potential for countries in other profiles to learn from these examples and aim to replicate successful health system strategies.

However, we stand to learn much from countries outside the Goldilocks zone. Insights can be taken from countries in all profiles in relation to the mitigation of the health impacts of climate change and the reduction of health system emissions. These efforts have been, and will continue to be, undertaken in the context of a country’s matrix of vulnerability, HDI, and emissions, and can therefore serve as examples for other countries, including those outside the OECD, with similar characteristics. That is to say, we cannot expect Vulnerable low emitters (Profile 1) to immediately adopt strategies used by those in the Goldilocks zone (Profile 5). Instead, Vulnerable low emitters could look to neighbouring profiles to identify key areas for improvement, and may in turn have levels of performance or specific initiatives that are beneficial to others.

For example, Mexico was classed as Profile 1, a Vulnerable low emitter. Despite its low emissions per capita relative to other profiles, it is second-largest greenhouse gas emitter in Latin America and the Caribbean^25,26^. In response, Mexico has committed to several policy changes to address its carbon footprint^25^. Mexico’s geography renders it highly vulnerable to the impacts of climate change^27^, and thus its population will likely experience a heavy burden of climate-related impacts, including large increases in heat-related illness, vector borne diseases, and respiratory disease^27^. However, due to the country’s lower HDI, Mexico’s health system is less well-resourced to respond to these impacts, which is likely to exacerbate the burden of climate change on the system. It is here that Mexico, and other countries in Profile 1, have the biggest opportunity to improve their capacity to adapt to the health-related impacts of climate change. That is, Mexico’s chief focus might not necessarily be climate-specific health adaptation, but rather a broader goal of overall improvement in HDI.

To accomplish such strategies, countries in Profile 1 could look to those in the Middle of the road (Profile 3), which have a similar emissions profile and vulnerability to climate change, but a higher HDI. For example, Türkiye has similar emissions per capita to Mexico and is also highly vulnerable to the impacts of climate change^28^. However, in recent years, Türkiye demonstrates an upward trend in its HDI ranking, largely due to investment in public education and healthcare^29^ and a focus on digital transformation^30^ Despite this, the country nevertheless faces challenges, with regional disparities in access to education and healthcare services^29^ As such, the policies and strategies implemented in Türkiye, and those used by other countries in Profile 3, could be examined and adapted by countries in Profile 1, to sharpen their capacity and focus to manage the health-related impacts of climate change.

## Conclusion

Our study provides a benchmarking system comparing OECD countries’ and their health systems’ capacities to mitigate their GHG emissions and adapt to climate change. By examining associations across GHGs, climate vulnerability and human development, we identified five distinct profiles of countries, each representing challenges and opportunities in health system resilience and sustainability. The journey to net zero is not destined to go smoothly, but we observe that OECD countries are, to a greater or lesser extent, making progress. Countries in the Goldilocks zone demonstrate a balance of low emissions, higher development and reduced vulnerability, enabling stronger health system performance. In contrast, countries within the Vulnerable low emitters group face challenges such as higher climate vulnerabilities with lower resources constraining their mitigation and adaptation efforts. An analysis of differences between countries on climate factors and health systems performance offer opportunities for increased understanding and improvement. Importantly, by providing a benchmark, our analyses enable the monitoring of progress over time, providing a new tool to track the impacts of mitigation and adaptation policies and strategies at country level.

## Electronic supplementary material

Below is the link to the electronic supplementary material.

Supplementary Material 1

## Data Availability

All datasets used in this study are publicly available via the OECD (https://doi.org/10.1787/env-data-en), ND-GAIN (https://gain.nd.edu/our-work/country-index/download-data/), and United Nations https://hdr.undp.org/data-center/human-development-index#/indicies/HDI). Additional details related to methodology and analysis are available in the supplementary materials provided or upon request from the corresponding author.
