# Supplementary material for: Health system performance on greenhouse gas emissions, climate change and development status in 38 OECD countries
Source: Sci Rep. 2025 Feb 11;15:5101. doi: 10.1038/s41598-025-89485-0 (PMC11814310; doi:10.1038/s41598-025-89485-0)
Supplement: Supplementary file 1 — Supplementary Material 1 [file 41598_2025_89485_MOESM1_ESM.pdf]

12. SUPPLEMENTARY MATERIALS

Table S1: Post Hoc Comparison of Group Differences in Health Risk Factors

Group 1	Group 2	Mean Difference	Std. Error	p-value	95% Confidence Interval
Vulnerable low emitters	Vulnerable aspiring	0.62	0.35	0.85	-0.43, 1.68
Vulnerable low emitters	Middle of the road	1.53	0.31	<0.001**	0.59, 2.47
Vulnerable low emitters	Robust aspiring	0.84	0.33	0.15	-0.14, 1.81
Vulnerable low emitters	Goldilocks zone	0.82	0.31	0.13	-0.12, 1.76
Vulnerable aspiring	Vulnerable emitters	-0.62	0.35	0.85	-1.68, 0.43
Vulnerable aspiring	Middle of the road	0.91	0.26	0.01**	0.13, 1.69
Vulnerable aspiring	Robust aspiring	0.21	0.27	1.00	-0.61, 1.04
Vulnerable aspiring	Goldilocks zone	0.20	0.26	1.00	-0.58, 0.98
Middle of the road	Vulnerable emitters	-1.53	0.31	<0.001**	-2.47, -0.59
Middle of the road	Vulnerable aspiring	-0.91	0.26	0.01**	-1.69, -0.13
Middle of the road	Robust aspiring	-0.70	0.22	0.04*	-1.37, -0.03
Middle of the road	Goldilocks zone	-0.71	0.2	0.01*	-1.33, -0.1
Robust aspiring	Vulnerable emitters	-0.84	0.33	0.15	-1.81, 0.14
Robust aspiring	Vulnerable aspiring	-0.21	0.27	1.00	-1.04, 0.61
Robust aspiring	Middle of the road	0.70	0.22	0.04*	0.03, 1.37
Robust aspiring	Goldilocks zone	-0.02	0.22	1.00	-0.69, 0.66
Goldilocks zone	Vulnerable emitters	-0.82	0.31	0.13	-1.76, 0.12
Goldilocks zone	Vulnerable aspiring	-0.20	0.26	1.00	-0.98, 0.58
Goldilocks zone	Middle of the road	0.71	0.2	0.01**	0.1, 1.33
Goldilocks zone	Robust aspiring	0.02	0.22	1.00	-0.66, 0.69

Table S2: Post Hoc Comparison of Group Differences in Healthcare System Resources

Group 1	Group 2	Mean Difference	Std. Error	p-value	95% Confidence Interval
Vulnerable low emitters	Vulnerable aspiring	-1.78	0.31	<0.001**	-2.70, -0.86
Vulnerable low emitters	Middle of the road	-1.09	0.27	<0.001**	-1.91, -0.27
Vulnerable low emitters	Robust aspiring	-1.56	0.28	<0.001**	-2.41, -0.70
Vulnerable low emitters	Goldilocks zone	-1.75	0.27	<0.001**	-2.57, -0.93
Vulnerable aspiring	Vulnerable emitters	1.78	0.31	<0.001**	0.86, 2.70
Vulnerable aspiring	Middle of the road	0.7	0.23	0.04*	0.02, 1.38
Vulnerable aspiring	Robust aspiring	0.23	0.24	1.00	-0.49, 0.94
Vulnerable aspiring	Goldilocks zone	0.04	0.23	1.00	-0.64, 0.71
Middle of the road	Vulnerable emitters	1.09	0.27	<0.001**	0.27, 1.91
Middle of the road	Vulnerable aspiring	-0.7	0.23	0.04*	-1.38, -0.02
Middle of the road	Robust aspiring	-0.47	0.19	0.21	-1.06, 0.11
Middle of the road	Goldilocks zone	-0.66	0.18	0.01**	-1.20, -0.12
Robust aspiring	Vulnerable emitters	1.56	0.28	<0.001**	0.70, 2.41
Robust aspiring	Vulnerable aspiring	-0.23	0.24	1.00	-0.94, 0.49
Robust aspiring	Middle of the road	0.47	0.19	0.21	-0.11, 1.06
Robust aspiring	Goldilocks zone	-0.19	0.19	1.00	-0.78, 0.39
Goldilocks zone	Vulnerable emitters	1.75	0.27	<0.001**	0.93, 2.57
Goldilocks zone	Vulnerable aspiring	-0.04	0.23	1.00	-0.71, 0.64

Goldilocks zone	Middle of the road	0.66	0.18	0.01**	0.12, 1.20
Goldilocks zone	Robust aspiring	0.19	0.19	1.00	-0.39, 0.78

Table S3: Post Hoc Comparison of Group Differences in Health Status

Group 1	Group 2	Mean Difference	Std. Error	p-value	95% Confidence Interval
Vulnerable low emitters	Vulnerable aspiring	-1.29	0.43	0.05*	-2.59, 0.01
Vulnerable low emitters	Middle of the road	-0.45	0.38	1.00	-1.60, 0.71
Vulnerable low emitters	Robust aspiring	-1.42	0.4	0.01**	-2.63, -0.22
Vulnerable low emitters	Goldilocks zone	-1.40	0.38	0.01**	-2.56, -0.25
Vulnerable aspiring	Vulnerable emitters	1.29	0.43	0.05*	-0.01, 2.59
Vulnerable aspiring	Middle of the road	0.84	0.32	0.12	-0.11, 1.80
Vulnerable aspiring	Robust aspiring	-0.13	0.34	1.00	-1.15, 0.88
Vulnerable aspiring	Goldilocks zone	-0.11	0.32	1.00	-1.07, 0.85
Middle of the road	Vulnerable emitters	0.45	0.38	1.00	-0.71, 1.60
Middle of the road	Vulnerable aspiring	-0.84	0.32	0.12	-1.80, 0.11
Middle of the road	Robust aspiring	-0.98	0.27	0.01**	-1.80, -0.15
Middle of the road	Goldilocks zone	-0.96	0.25	0.01**	-1.71, -0.20
Robust aspiring	Vulnerable emitters	1.42	0.4	0.01**	0.22, 2.63
Robust aspiring	Vulnerable aspiring	0.13	0.34	1.00	-0.88, 1.15
Robust aspiring	Middle of the road	0.98	0.27	0.01**	0.15, 1.80
Robust aspiring	Goldilocks zone	0.02	0.27	1.00	-0.80, 0.85
Goldilocks zone	Vulnerable emitters	1.40	0.38	0.01**	0.25, 2.56
Goldilocks zone	Vulnerable aspiring	0.11	0.32	1.00	-0.85, 1.07
Goldilocks zone	Middle of the road	0.96	0.25	0.01**	0.20, 1.71
Goldilocks zone	Robust aspiring	-0.02	0.27	1.00	-0.85, 0.80

Table S4: Post Hoc Comparison of Group Differences in Quality of Healthcare

Group 1	Group 2	Mean Difference	Std. Error	p-value	95% Confidence Interval
Vulnerable low emitters	Vulnerable aspiring	-1.43	0.39	0.01**	-2.59, -0.26
Vulnerable low emitters	Middle of the road	-0.75	0.34	0.37	-1.79, 0.29
Vulnerable low emitters	Robust aspiring	-1.37	0.36	0.01**	-2.44, -0.29
Vulnerable low emitters	Goldilocks zone	-1.42	0.34	<0.001**	-2.46, -0.38
Vulnerable aspiring	Vulnerable emitters	1.43	0.39	0.01**	0.26, 2.59
Vulnerable aspiring	Middle of the road	0.67	0.29	0.24	-0.19, 1.53
Vulnerable aspiring	Robust aspiring	0.06	0.3	1.00	-0.85, 0.97
Vulnerable aspiring	Goldilocks zone	0.01	0.29	1.00	-0.85, 0.87
Middle of the road	Vulnerable emitters	0.75	0.34	0.37	-0.29, 1.79
Middle of the road	Vulnerable aspiring	-0.67	0.29	0.24	-1.53, 0.19
Middle of the road	Robust aspiring	-0.61	0.25	0.18	-1.35, 0.13
Middle of the road	Goldilocks zone	-0.67	0.23	0.06	-1.35, 0.01
Robust aspiring	Vulnerable emitters	1.37	0.36	0.01**	0.29, 2.44
Robust aspiring	Vulnerable aspiring	-0.06	0.3	1.00	-0.97, 0.85
Robust aspiring	Middle of the road	0.61	0.25	0.18	-0.13, 1.35

Robust aspiring	Goldilocks zone	-0.05	0.25	1.00	-0.79, 0.69
Goldilocks zone	Vulnerable emitters	1.42	0.34	<0.001**	0.38, 2.46
Goldilocks zone	Vulnerable aspiring	-0.01	0.29	1.00	-0.87, 0.85
Goldilocks zone	Middle of the road	0.67	0.23	0.06	-0.01, 1.35
Goldilocks zone	Robust aspiring	0.05	0.25	1.00	-0.69, 0.79

Table S5: Post Hoc Comparison of Group Differences in Access to Healthcare

Group 1	Group 2	Mean Difference	Std. Error	p-value	95% Confidence Interval
Vulnerable low emitters	Vulnerable aspiring	-1.72	0.34	<0.001**	-2.75, -0.69
Vulnerable low emitters	Middle of the road	-0.61	0.31	0.53	-1.53, 0.31
Vulnerable low emitters	Robust aspiring	-1.58	0.32	<0.001**	-2.54, -0.62
Vulnerable low emitters	Goldilocks zone	-1.68	0.31	<0.001**	-2.60, -0.76
Vulnerable aspiring	Vulnerable emitters	1.72	0.34	<0.001**	0.69, 2.75
Vulnerable aspiring	Middle of the road	1.11	0.25	<0.001**	0.35, 1.87
Vulnerable aspiring	Robust aspiring	0.14	0.27	1.00	-0.66, 0.95
Vulnerable aspiring	Goldilocks zone	0.04	0.25	1.00	-0.72, 0.80
Middle of the road	Vulnerable emitters	0.61	0.31	0.53	-0.31, 1.53
Middle of the road	Vulnerable aspiring	-1.11	0.25	<0.001**	-1.87, -0.35
Middle of the road	Robust aspiring	-0.97	0.22	<0.001**	-1.62, -0.31
Middle of the road	Goldilocks zone	-1.07	0.2	<0.001**	-1.67, -0.46
Robust aspiring	Vulnerable emitters	1.58	0.32	<0.001**	0.62, 2.54
Robust aspiring	Vulnerable aspiring	-0.14	0.27	1.00	-0.95, 0.66
Robust aspiring	Middle of the road	0.97	0.22	<0.001**	0.31, 1.62
Robust aspiring	Goldilocks zone	-0.1	0.22	1.00	-0.76, 0.56
Goldilocks zone	Vulnerable emitters	1.68	0.31	<0.001**	0.76, 2.60
Goldilocks zone	Vulnerable aspiring	-0.04	0.25	1.00	-0.80, 0.72
Goldilocks zone	Middle of the road	1.07	0.2	<0.001**	0.46, 1.67
Goldilocks zone	Robust aspiring	0.1	0.22	1.00	-0.56, 0.76

Table S6: Post Hoc Comparison of Group Differences in COVID-19 Factors

Group 1	Group 2	Mean Difference	Std. Error	p-value	95% Confidence Interval
Vulnerable low emitters	Vulnerable aspiring	-1.43	0.47	0.05*	-2.85, -0.01
Vulnerable low emitters	Middle of the road	-0.51	0.42	1.00	-1.78, 0.75
Vulnerable low emitters	Robust aspiring	-1.1	0.44	0.17	-2.42, 0.21
Vulnerable low emitters	Goldilocks zone	-0.98	0.42	0.26	-2.25, 0.29
Vulnerable aspiring	Vulnerable emitters	1.43	0.47	0.05*	0.01, 2.85
Vulnerable aspiring	Middle of the road	0.92	0.35	0.13	-0.13, 1.96
Vulnerable aspiring	Robust aspiring	0.33	0.37	1.00	-0.78, 1.44
Vulnerable aspiring	Goldilocks zone	0.45	0.35	1.00	-0.60, 1.50
Middle of the road	Vulnerable emitters	0.51	0.42	1.00	-0.75, 1.78
Middle of the road	Vulnerable aspiring	-0.92	0.35	0.13	-1.96, 0.13
Middle of the road	Robust aspiring	-0.59	0.3	0.59	-1.49, 0.32
Middle of the road	Goldilocks zone	-0.47	0.28	1.00	-1.29, 0.36

Robust aspiring	Vulnerable emitters	1.1	0.44	0.17	-0.21, 2.42
Robust aspiring	Vulnerable aspiring	-0.33	0.37	1.00	-1.44, 0.78
Robust aspiring	Middle of the road	0.59	0.3	0.59	-0.32, 1.49
Robust aspiring	Goldilocks zone	0.12	0.3	1.00	-0.78, 1.02
Goldilocks zone	Vulnerable emitters	0.98	0.42	0.26	-0.29, 2.25
Goldilocks zone	Vulnerable aspiring	-0.45	0.35	1.00	-1.50, 0.60
Goldilocks zone	Middle of the road	0.47	0.28	1.00	.-0.36, 1.29
Goldilocks zone	Robust aspiring	-0.12	0.3	1.00	-1.02, 0.78
